# Interaction between Treg Apoptosis Pathways, Treg Function and HLA Risk Evolves during Type 1 Diabetes Pathogenesis

**DOI:** 10.1371/journal.pone.0036040

**Published:** 2012-04-26

**Authors:** Sanja Glisic, Parthav Jailwala

**Affiliations:** 1 Department of Pediatrics, Max McGee National Research Center for Juvenile Diabetes, Human and Molecular Genetics Center, Medical College of Wisconsin, Children's Hospital of Wisconsin, Milwaukee, Wisconsin, United States of America; 2 Advanced Biomedical Computing Center, SAIC-Frederick/NCI-Frederick, Bethesda, Maryland, United States of America; NIH-NCI, United States of America

## Abstract

We have previously reported increased apoptosis of regulatory T cells (Tregs) in recent-onset Type 1 Diabetes subjects (RO T1D) in the honeymoon phase and in multiple autoantibody-positive (Ab+) subjects, some of which are developing T1D. We have also reported that increased Treg apoptosis was associated with High HLA risk and that it subsided with cessation of honeymoon period. In this report, we present results generated using genetics, genomics, functional cell-based assays and flow cytometry to assess cellular changes at the T-cell level during T1D pathogenesis. We measured *ex vivo* Treg apoptosis and Treg function, surface markers expression, expression of HLA class II genes, the influence of HLA risk on Treg apoptosis and function, and evaluated contribution of genes reported to be involved in the apoptosis process. This integrated comprehensive approach uncovered important information that can serve as a basis for future studies aimed to modulate Treg cell responsiveness to apoptotic signals in autoimmunity. For example, T1D will progress in those subjects where increased Treg apoptosis is accompanied with decreased Treg function. Furthermore, Tregs from High HLA risk healthy controls had increased Treg apoptosis levels and overexpressed FADD but not Fas/FasL. Tregs from RO T1D subjects in the honeymoon phase were primarily dying through withdrawal of growth hormones with contribution of oxidative stress, mitochondrial apoptotic pathways, and employment of TNF-receptor family members. Ab+ subjects, however, expressed high inflammation level, which probably contributed to Treg apoptosis, although other apoptotic pathways were also activated: withdrawal of growth hormones, oxidative stress, mitochondrial apoptosis and Fas/FasL apoptotic pathways. The value of these results lie in potentially different preventive treatment subjects would receive depending on disease progression stage when treated.

## Introduction

Type 1 Diabetes (T1D) is one of the most prevalent autoimmune chronic diseases in children with a raising incidence of ∼3% annually [1], [2]. T1D has a complex etiology as it is influenced by multiple genetic and environmental risk factors. The inherited genetic factors influence both susceptibility and resistance to the disease [3], [4]. The genetics of T1D has a long history of studies evaluating candidate genes for association with disease status using either case-control or family-based studies. These studies revealed that the major susceptibility genetic locus for T1D lies in the major histocompatibility complex (MHC) region referred as IDDM1 [5]. The MHC region is located on the short arm of chromosome 6 (6p21.3) spanning an interval of ∼4 Mb. Although the interval contains over 200 expressed genes [6], candidate gene studies have implicated human histocompatibility antigens (*HLA*) as being the primary T1D susceptibility locus [7], [8]. The importance of the *HLA* region in the determination of T1D risk was discovered in the 1970s [9]. Early family studies comparing disease concordance in monozygotic twins and HLA-matched siblings established the significance of HLA region genes accounting for about 50% of the genetic risk [10].

Although extremely polymorphic [11], the major susceptibility for T1D has been mapped to the HLA class II genes *HLA-DQB1*, *HLA-DQA1* and *HLA-DRB1* [12], [13]. The three genes have been shown to independently contribute to T1D susceptibility with the strongest single effect coming from HLA-DQB1 [3]. The molecular basis for the association between at risk alleles and T1D is still not clear. The *HLA* gene products are known to be heterodimeric transmembrane glycoproteins that involve non-covalently associated α- and β-chains, each having two extracellular domains (α1 and α2 or β1 and β2). These molecules are expressed on B lymphocytes, macrophages, and other cells of the immune system termed antigen-presenting cells (APC) whose role is to present antigens to T and B cells. Qualitative differences in antigen presentation between the predisposing and the protective DQ molecules coded by different alleles have been demonstrated in functional studies, and these molecules are shown to contribute to differences in the ability to activate autoreactive T cells involved in pancreatic beta-cell destruction [14], [15], suggesting that *HLA* genes have an important role in antigen-specific regulation of T-cell activation. However, HLA class II genes are also signaling molecules leading to a variety of cellular responses including cell-cell adhesion, proliferation, differentiation and apoptosis [16], [17], [18], [19]. CD4 T cells recognize *HLA* class II gene products while CD8 T cells recognize *HLA* class I gene products. Variations in *HLA* genes could, therefore, account for differential transcriptional activities and quantities of mRNA of genes involved in the activation/signal transduction. A quantitative hierarchy of DRB1 mRNA in healthy individuals has been observed for different alleles (DRB1*03>DRB1*04>DRB1*01>DRB1*08) [20]. Similar quantitative hierarchy has been observed for *DQA1* (DQA1*0301>DQA1*0101>DQA1*0501) [21] and *DQB1* genes (DQB1*0301>DQB*0501>DQB1*0602) [22]. Over 90% of Caucasian diabetic subjects possess one of susceptibility haplotypes HLA-DR4-DQA1*0301-DQB1*0302 or HLA-DR3-DQA1*0501-DQB1*0201 or both [23], [24], [25]. The HLA-DR15-DQA1*0102-DQB1*0602 haplotype is protective, and rarely present in T1D subjects [26], [27], [28]. As polymorphisms in the *DR* and *DQ* genes appear to be of great biological importance suggesting their involvement in the etiology of the disease [4], HLA class II genes are considered to be the best genetic markers for T1D [29] currently available.

Our Wisconsin Family T1D Study involving non-related random healthy controls, recent-onset (RO) T1D, healthy autoantibody-positive (Ab+) siblings of T1D probands and longstanding (LS) T1D subjects, has allowed us to develop an *HLA DQA1/B1* haplotype risk assessment tool that recognizes susceptible (S), resistant (R), weakly protective (Y) and neutral haplotypes (X). When these *HLA DQA1/B1* haplotypes are combined into haplogenotypes (please see table S1, and in ref [30]), they provide information about HLA risk status with four categories: low, moderate, high and very high risk [30]. These four categories we further condensed into two-tier system, Low and High HLA risk for easier comparison between T1D-related subject groups. We found that there is a significant association of high HLA risk status and T1D incidence in Wisconsin cohort, as >90% of affected subjects possess High HLA risk haplotypes (Table 1). Our results are consistent with previously published studies [24], [25], [31]. Earlier, we have reported a correlation between High HLA risk haplotypes and increased Treg apoptosis [30]. The present cross-sectional study was designed to: 1) validate this result in larger sample of subjects, 2) presents additional lines of evidence showing an association between HLA gene expression and Treg apoptosis and function, 3) assess change in Treg function during T1D progression, and 4) assess activation of apoptosis pathways in T cells.

**Table 1:** Demographic data of studied population.

	Random Control	Recent-onset T1D	Auto-Ab+	Longstanding T1D	p value
**Number of subjects**	35	29	23	20	NS
**Average time after diagnosis (years)**	N/A*	0.58±0.16	N/A*	2.62±0.64	0.000871
**Gender (% female)**	54.3	42.8	54.5	42.8	NS
**BMI at recruitment**	22.75±0.73	17.47±0.76	20.90±1.20	18.51±1.00	<0.001
**Age at diagnosis (years)**	N/A*	10.85±0.74	N/A*	9.63±0.77	NS
**Age at recruitment (years)**	30.22±2.75	12.05±0.91	27.58±3.16	12.01±1.13	<0.0001
**Glucose at recruitment (mg/dl^−1^)**	89.26±1.91	163.31±14.14	84.41±1.14	162.87±12.74	<0.0001
**HbA1c (%)**	N/A	7.00±0.19	N/A	6.97±0.32	NS
**Insulin dose at recruitment (U/kg/day)**	N/A*	0.40±0.03	N/A*	0.62±0.05	<0.0001
**High HLA risk (%)**	37.1	89.6	69.6	90	<0.001
**Treg apoptosis (%)**	3.84±0.49	15.52±2.66	5.8±0.74	4.04±0.45	<0.0001

All data presented as (mean value)±SE; N/A – not available;

* - not applicable.

Our results show that T1D will progress in those subjects where increased Treg apoptosis is accompanied with decreased Treg function. We also show that different apoptosis pathways are prevalent during T1D progression. The value of these results lie in potentially different preventive treatment subjects would receive depending on disease progression stage when treated.

## Materials and Methods

### Healthy subjects

One hundred and seven subjects were recruited through the diabetes program at Children's Hospital of Wisconsin. Recent - onset T1D subjects (after stabilization on exogenous insulin but within 10 months of diagnosis; n = 29) were recruited through the diabetes program at Children's Hospital of Wisconsin. Diabetes was defined according to World Health Organization criteria and included blood glucose levels of >200 mg/dl with symptoms confirmed by a physician [32]. Healthy control (n = 35) subjects were recruited by posting flyers in Children's Hospital of Wisconsin and the Medical College of Wisconsin. The control criteria comprised fasting blood glucose of <100 mg/dl, no familial history of any autoimmune disorder, and lack of islet autoantibodies. We also recruited autoantibody-positive siblings of T1D probands not included in this study (n = 23) and longstanding (LS) T1D subjects (n = 20). All study subjects were free of known infection at the time of sample collection. At the time of each visit, the following clinical measurements were taken: HbA1c, glucose level, height, weight and BMI (subject characteristics are shown in Table 1). DNA was collected for HLA typing and presence of autoantibodies was measured from peripheral blood. The research protocol was approved by the Institutional Review Board (IRB) of the Children's Hospital of Wisconsin and participants and/or their parents (guardians) provided written informed consent.

### PBMC and FACS cell isolation

Peripheral blood mononuclear cells (PBMC) were collected using vacutainers with ACD solution B of trisodium citrate and isolation was done using Ficoll-Hypaque (Amersham Pharmacia, Uppsala, Sweden) gradient density centrifugation according to the recommended protocol. The PBMC were counted and stained with a cocktail of fluorochrome conjugated monoclonal antibodies in PBS (APC-aCD4 (clone RPA-T4), APC-Cy7-aCD25 or PE-aCD25 (clone M-A251), FITC-aCD14 (clone M5E2), FITC-aCD32 (clone FL18.26), FITC-aCD116 (cloneM5D12), PE-Cy7-aCD8 (clone RPA-T8) all from BD Pharmingen, San Diego, CA) and sorted on a FACSAria (BD Biosciences, San Diego, CA). Using the same FACS isolation protocol described earlier [33], we collected the top 1% of CD4^+^CD25^high^ T-cells as Tregs. This additional stringency of collecting just the top 1% of CD4^+^CD25^high^ cells as Tregs across subject groups ensured removal of most of the activated CD25low T cells. CD4+ T cells were further gated as CD4+25−, CD4+25low and CD4+CD25^high^ using the Fluorochrome Minus One (FMO) method, which allowed for a more precise definition of cells having fluorescence above the background level. The cells expressing low levels of CD25 were collected and defined as CD4+CD25low T cells. Based on performed assays, our isolation protocol generated Tregs that maintained high level of sustained FOXP3 expression associated with phenotypic and functional stability. HLA DQ-PE (clone 1a3) antibody recognizing all DQ alleles [34] was purchased from Leinco Technologies, Inc (St.Louis, MO).

### Cell culture and Suppression assay

CD4^+^CD25− and CD4+CD25low T-cells (2.5×10^4^ cells/well) were cultured in RPMI 1640 media supplemented with 2 mM L-glutamine, 5 mM HEPES, 100 U/µg/ml penicillin/streptomycin, 0.5 mM sodium pyruvate and 10% human AB serum. Cells were stimulated with aCD3 coated beads (1 µg/ml, 3 beads/cell) in U-bottom 96-well plates (Costar) in the presence of the same number of irradiated autologous PBMC for 3 days. For the suppression assays Treg cells were co-cultured with responder T cells at a 1∶10 ratio (Treg∶Tresponder) using the same stimuli. Cells were pulsed with 1 µCi of [^3^H] thymidine (Amersham Pharmacia Biotech) and harvested after 16 hours. The cpm per well was determined with a scintillation counter (Top Count NXT, Packard). The percentage of suppression was calculated as {(s−c)/s}×100%, where s = cpm in single culture and c = cpm in co-culture.

### HLA genotyping

Genotyping of both HLA-DQA1 and HLA-DQB1 was performed by direct sequencing of the polymorphic regions of each gene. For HLA-DQA1, Exon 2 was sequenced, yielding a low-resolution (2-digit) typing result. For HLA-DQB1, Exon 2 was sequenced using SeCore DQB1 Locus Sequencing Kits (Invitrogen, Brown Deer, WI). This method yields high-intermediate (4-digit resolution) of HLA-DQB1. HLA-DQA1-DQB1 haplotypes and 4-digit resolution of HLA-DQA1 were then inferred using Caucasian frequencies as reported by Klitz, et al. [35].

### Apoptosis assay

Apoptosis was measured in CD4+CD25−, CD4+CD25+low and CD4+CD25+^high^ T cells immediately after FACS sorting for baseline apoptosis levels and before exposure to any stimulation. The cells were stained in the dark with 250 nM YOPRO1 (Molecular Probes, Eugene, OR) for 20 min and then 250 ng 7AAD (BD Biosciences) was added 10 min before acquiring at least 10,000 events on LSRII FACS (BD Bioscience) machine. The thresholds for both YOPRO1 and 7AAD were determined based on the forward and side scatter properties of the naïve T cells. Apoptosis was measured as the percentage of apoptotic cells (YOPRO1^+^/7AAD^−^) among live cells (all 7AAD^−^ cells comprising both YOPRO1^+^ and YOPRO1^−^ cells)

### PCR array of naïve and Treg cells

Apoptosis PCR array (SABiosciences, Frederick, MD) of naïve and Treg cells was done on a subset of subjects involved in this study: unaffected subjects in High HLA control group (n = 8), unaffected subjects in Low HLA control group (n = 5), unaffected multiple Ab+ subject group (n = 4) and affected RO T1D (n = 4). Apoptosis PCR array used in this study was a 384-well (4×96) plate consisting of 84 key genes involved in programmed cell death. There are 12 other wells set up for quality controls, for example checking genomic DNA contamination, reverse transcription efficacy and PCR array reproducibility. The plate set up used in this study was done in pairs for every subject (naïve and Tregs from the same subject) and, whenever possible, in pairs for control and RO T1D/multiple Ab+ subjects. Gene expression of Tregs from every subject was then normalized with gene expression of autologous naïve T cells to account for expression induced by common factors coming from, for example, T cell lineage commitment or the procedure of cell isolation. Such normalized values were then compared between groups. The analysis of gene expression was done through comparison to Low HLA risk healthy control subject group.

### Real-Time PCR analysis

The second portion of isolated total RNA was converted to cDNA using the QuantiTect® Reverse Transcription Kit (QIAGEN, Valencia, CA). Real-time PCR was then performed using the QuantiTect® SYBR Green PCR Kit (QIAGEN) on an ABI Prism 7900HT Sequence Detection System machine using SDS software (Applied Biosystems, Foster City, CA). Manufacturer protocols were followed for all procedures. RNA expression was quantitated relative to 18S RNA expression. mRNA gene-expression was quantitated relative to GAPDH mRNA expression. Using the Oligo 6 software (Molecular Biology Insights), primer sets for each gene were designed towards the same region of cDNA that was represented by the probe sets on the Affymetrix GeneChip arrays, and RT-PCR validation was performed.

### *In vitro* inhibition of Treg apoptosis

Tregs isolated from random healthy control, RO T1D, Ab+ subjects and LS T1D subjects were treated either with soluble FasL (1/40 dilution or 600 ng/ml) or with plate-bound aCD3 (clone UCHT1, Ancell) at high concentration (20 µg/ml) causing activation-induced cell death (AICD). Separate cell aliquots were pre-treated with Z-DEVD (caspase 3 inhibitor) or with Ac-IETD (caspase 8 inhibitor) for 30 minutes before exposure to stimulation with either soluble FasL or to AICD agent and apoptosis was measured using YOPRO1/7AAD, as described above.

### Statistics

The Mann-U-Whitney and Tukey-Kramer tests were used to compare generated results between clinical groups, with p value ≤0.05 considered significant. GraphPad software was used for data presentation. We also performed Kruskal-Wallis test in addition to a one-way ANOVA. Linear regression model was used for association studies of Treg functional measurements (*in vitro* suppression of proliferation of responder T cells) and HLA risk for T1D.

Quality control and normalization for microarray data was done as previously described [36]. Briefly, inspected RNA degradation across all arrays showed no significant differences in the degradation patterns. Quality control was done through visual inspection of each microarray scan for irregularities, and the whole microarray set was assessed using the ‘affyQCreport’ package from the Bioconductor project (Halling *et al*, 2006). The quality of the data was ascertained by inspecting various plots. Raw expression values were normalized across all 27 samples by computing the Robust Multichip Average (RMA) directly from the Affymetrix .CEL files (Irrizary et al. 2003), generating expression measure on the log base 2 scale.

Gene expression differences between RO T1D and control samples were captured earlier using Affymetrix GeneChip human genome U133 Plus 2.0 arrays. The experimental design, quality control procedure, detailed statistical analysis and results are described in [36].

## Results and Discussion

This study involved subjects belonging to several cohorts aimed at capturing different points of T1D progression, in an effort to increase our ability to detect positive correlation between generated results with T1D pathogenesis. These cohorts included: unrelated healthy controls (no T1D), siblings of probands (not included in this study) possessing multiple Ab+ (in a stage of developing T1D), recent-onset (RO) T1D (experienced T1D onset, but in the honeymoon phase when endogenous insulin production temporarily improves) and longstanding (LS) T1D subjects (fully dependent on exogenous insulin) (Table 1). Each of these subject groups was also tested for correlation of the results relative to HLA risk. For this comparison, we gathered data capturing changes at the genetic, genomic or transcriptional as well as at protein and functional level in different cell subsets, isolated by Fluorescent Activated Cell Sorting (FACS). Thus, we present here several lines of evidence that confirm differences in monitored traits between Low and High HLA risk subjects when divided according to T1D status.

### HLA risk associated with Treg apoptosis levels

**Figure 1:**
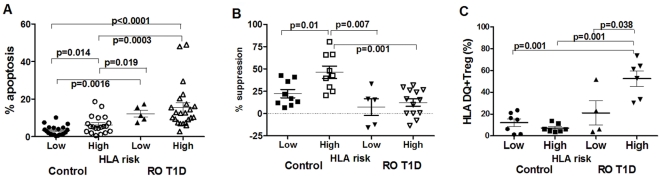
Treg apoptosis, function and surface HLA DQ relative to HLA risk in healthy control and RO T1D subjects. A) Healthy High HLA risk control subjects show significantly increased Treg apoptosis levels (ANOVA F = 10.24 df(3,64), p<0.0001). Detailed significance presented in the figure done using Mann-U-Whitney test. However, RO T1D subjects that succumbed disease show significantly increased Treg apoptosis levels independently on HLA risk. B) High HLA risk healthy control subjects show significantly increased suppressive function of their Tregs compared to Low HLA risk controls (Mann-U-Whitney test, p = 0.01), while that association was lost in RO T1D group (Mann-U-Whitney, p = 0.69). C) RO T1D subjects with High HLA risk haplotypes show significantly higher surface DQ expression compared to Low HLA risk both RO T1D and healthy control subjects while High HLA risk healthy controls express the least number of cells with surface HLA DQ (ANOVA F = 14.62 df(3,20), p<0.0001). Detailed comparisons was done using Mann-U-Whitney test. Values are presented with standard errors.

As HLA has been recognized as a major genetic risk factor, subject groups involved in this study were partitioned into two HLA risk groups (Low and High) according to the scheme we have developed earlier [30]. We have linked Treg apoptosis with HLA risk for T1D in our previous study [30] and in this study we verify the trend in a larger sample of subjects (Figure 1A). Partitioning of healthy controls and RO T1D subjects on Low and High HLA risk groups (High involving Moderate, High and Very High HLA risk groups), reveals increased Treg apoptosis levels in High HLA risk control subjects when compared to their Low HLA risk counterparts, while Treg apoptosis difference between the two HLA risk groups has not been seen in RO T1D subject group. This suggests that in healthy control subjects there is an association of HLA risk with Treg apoptosis. In disease state, however, T1D progression overrides this association, increasing Treg apoptosis levels further independently of HLA risk. High HLA risk status was associated with increased Treg suppressive function in Control group, most likely offering an explanation of why High risk control subjects do not succumb the disease. High HLA risk RO T1D group had significantly lower Treg function compared to High HLA risk healthy controls (Figure 1B). In a search for better understanding the association of HLA and Tregs, we have also measured frequency of healthy control and RO T1D subjects' Tregs expressing surface HLA DQ molecules (Figure 1C). Significantly higher frequency of Tregs from High HLA risk RO T1D subjects expressed surface DQ alleles compared to Tregs from High HLA risk healthy control subjects (p = 0.001). It has been acknowledged that, when activated, T cells also express HLA molecules on their surface [37]. The *HLA DQ* expression on Tregs implies potential antigen presentation to other T cells [38], [39], [40] encouraging belief that T-T cell interactions play an important role in the immune response [41].

**Figure 2:**
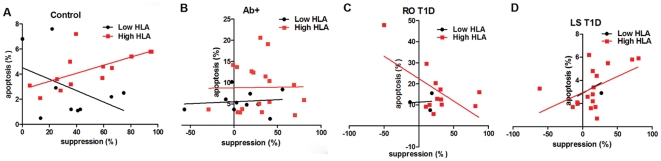
Changes in correlation of HLA risk with Treg apoptosis and Treg function in subject cohorts representing stages of T1D development. In High HLA risk both healthy control and LS T1D subjects, increased Treg apoptosis significantly correlated with increased Treg function (p = 0.017 and p = 0.042, respectively). High HLA risk RO T1D subjects showed opposite correlation: increased Treg apoptosis was correlated with decreased Treg function (p = 0.027). Multiple Ab+ subjects did not show correlation between Treg apoptosis and function in High HLA risk group (p = 0.95). Although some trends were present, Low HLA risk groups did not show correlation with Treg apoptosis and function.

Putting together HLA risk with Treg apoptosis and function in four different subject cohorts as representative of stages during T1D development, has shown changes in their correlation (Figure 2). Increased Treg suppressive function directly correlated with Treg apoptosis up to 6% in High HLA risk healthy control and LS T1D subject groups. The opposite was true for RO T1D, where higher suppressive function in High HLA risk RO T1D subjects correlated with lower Treg apoptosis. There was no significant correlation between the three factors in multiple Ab+ subjects. These findings prompted us to look into differences in HLA expression on Treg cells as well as mechanisms of Treg apoptosis.

### Differential expression of HLA class II genes might be impacted by HLA risk and/or disease progression

**Figure 3:**
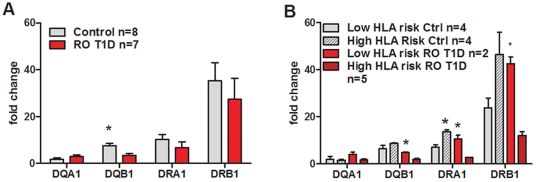
RT PCR verification of microarray data of HLA class II genes expression in Tregs isolated from Control and RO T1D subjects. A) HLA DR and DQ gene expression differences between control (n = 8) and RO T1D (n = 7) subjects. Only expression of DQB1 reached statistically significant difference (p = 0.045). B) Both Control (light grey bars) and RO T1D (red bars) subjects were divided according to HLA risk (see Table S1) and compared for expression of *HLA genes*. While High HLA risk (cross-pattern bars) control subjects had significantly higher expression of DRA1 compared to Low risk (empty bars, Mann-U-Whitney test, p = 0.045) Controls, Low HLA risk RO T1D subjects had higher expression of DRA1, but also DQB1 and DRB1 compared to corresponding High HLA risk RO T1D (Mann-U-Whitney test, p = 0.03, p = 0.03 and p = 0.01, respectively). Values are presented with standard errors.

We conducted gene expression profiling of un-manipulated, FACS isolated *ex vivo* Treg cells in the two most clinically distinct subject groups, healthy Control (n = 15) and RO T1D subjects (n = 12) and using GeneChip® Human Genome U133 Plus 2.0 array (Affymetrix, Santa Clara, CA) (data not shown here but deposited as GEO in [36]). In this report, the primary discussion was on the expression of apoptosis genes across RO T1D and controls. Although downregulation of HLA Class II genes in RO T1D was also observed in the results, this observation was not discussed in that report. In the current report, we highlight this down-regulation of HLA genes (HLA DQA1, DQB1, DRA1, DRB1 with fold changes of −5.3, −1.9, −2.2 and −1.6, respectively) and validate those results by RT-PCR (Figure 3A and 3B). The goal here is to better understand processes involved in T1D pathogenesis relative to the expression of HLA genes. RO T1D subjects express lower levels of *HLA* genes class II compared to healthy control subjects, with *HLA DQB1* reaching significance (p = 0.04). When both subject groups were divided according to HLA risk using our simplified scheme (Low and High HLA risk), opposite trend was noticed for control and RO T1D subjects. Namely, High HLA risk healthy controls showed higher expression of *HLA DQB1*, *DRA1* and *DRB1* compared to Low HLA risk control group, while opposite was true for RO T1D. Low HLA risk RO T1D had significantly higher expression of the three *HLA* class II genes compared to High HLA risk RO T1D subjects (Figure 3B). The same trend of the *HLA* class II genes' expression was detected in CD25low T cells (data not shown), suggesting effect of disease on the expression of *HLA* genes in all T cells without exerting cell specificity.

Our observation of reduced overall expression of HLA DR and DQ molecules in freshly isolated Tregs from RO T1D brings up an interesting but yet unexplored aspect of ‘inducible’ HLA expression and its role in Treg phenotype and function. It is not completely clear if *HLA gene* expression, the frequency of T cells expressing HLA molecules or the magnitude of expressed HLA molecules on the surface of T cells is relevant to the pathogenesis of diabetes. *HLA* Class II genes are constitutively expressed only on some cells (for example, antigen-presenting cells), but there is evidence of inducible HLA expression in several other cells, including Tregs [42]. The fact that we detected co-ordinated down-regulation of several *HLA* genes in disease could be explained through changes in expression or function of one or more transcription factors common across all genes within each HLA group. Analysis of the promoter regions of *HLA* class II genes, with the search restricted to locate transcription factors common across four *HLA* class II genes (DRA1, DRB1, DQA1 and DQB1) showed two transcription factors (NFYA and NFAT) down-regulated in Tregs from T1D subjects compared to healthy control subjects (0.93-fold, p = 0.12 and 0.97-fold, p = 0.045, respectively). Although expression differences in fold change were subtle, downregulation of NFAT in RO T1D has reached significance (p = 0.045). Interestingly, NFYA, which is a transcription factor regulating both Class I and Class II genes (*HLA* class I genes were also downregulated, data not shown) is located in the 6p21.31 region, roughly 8.4MB centromeric to T1D-linked *HLA DQB1* gene. Indeed, it was reported that NFYA participates in regulation of class II genes in activation-specific manner [43]. Significantly downregulated NFAT expression in RO T1D versus healthy control subjects could offer an explanation for reduced HLA expression we report here and IL-2 deprivation we reported earlier [36], considering that NFAT is a downstream molecule in IL-2 signaling pathway. NFAT is transcription factor important in the lifecycle not only effector T cells (forming complex with AP-1) but also in Tregs (forming complex with Foxp3). Namely, crystal structure of an NFAT∶FOXP∶DNA complex reveals an extensive protein-protein interaction interface between NFAT and FOXP family of proteins [44]. Thus, by switching transcriptional partners, NFAT converts the acute T cell activation program into the suppressor program of Tregs [44]. There is evidence that HLA expression can be induced in an environment rich in inflammatory cytokines [45], [46], suggestive of type of environment in the islets of a person progressing towards total beta-cell destruction. Furthermore, as human T lymphocytes are one of cell types that express HLA class II molecules following activation, these molecules on activated T cells could either affect the activity of T cells or they could provide signals that modulate T-cell functioning. If allogeneic dendritic cells expressing HLA-DQ (not HLA-DR) stimulate naïve T cells, they will shift towards Th2 phenotype [46]. As both *HLA-DQ* and *HLA-DR* genes, latter known as an activation marker, are down-regulated in Tregs from T1D subjects, it is possible that in T1D subjects, Tregs in the periphery have an activation defect, due to which they fail to exert suppressive function. Similar problem could affect effector T cells, causing lower IL-2 production leading to IL-2 deprivation, increased Treg apoptosis and decreased Treg function. Indeed, we have noticed that both effector T cell subsets isolated from RO T1D subjects proliferated less compared to control subjects (data not shown). Beside the current study, few other studies have focused on the expression of HLA molecules in T cells of patients with T1D, reporting unequal expression of *HLA DQA1* and *DQB1* alleles in total peripheral PBMC of T1D patients [47], [48].

### Common and different apoptosis pathways activated during T1D pathogenesis

In the light of observations of increased Treg apoptosis in High HLA risk subjects compared to Low HLA risk group (Figure 1A), we were specifically interested in the expression of genes involved in apoptosis pathways with a goal to identify potential differences in activated apoptotic pathways associated with T1D pathogenesis. PCR Arrays were used to measure expression of 84 genes of known apoptotic pathways in un-manipulated *ex vivo* naïve and Treg cell subsets from High HLA risk Control subject group (n = 8), Low HLA risk control subject group (n = 5), RO T1D patients (n = 4) and multiple Ab+ subjects (n = 4). Gene expression of Tregs from every subject was normalized with gene expression of the same genes in autologous naïve T cells to account for expression induced by common factors coming from, for example, T cell lineage commitment or the procedure of cell isolation. Such normalized values were then compared between groups. The analysis of gene expression in each subject group (High HLA control, RO T1D, and multiple Ab+) was done through comparison to Low HLA risk healthy control subject group.

**Figure 4:**
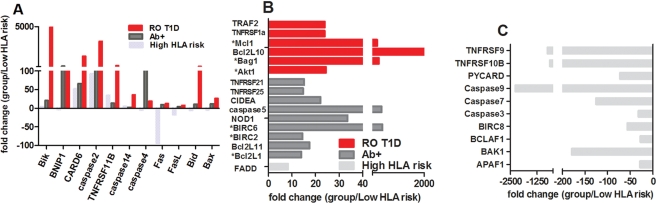
Differential gene expression in the three subject groups relative to Low HLA risk group. Ratio of gene expression between Tregs and CD25− from each subject was first obtained and averaged for each group before comparison to the Low HLA risk control subject group. A) fold change genes expression in the High HLA risk healthy control (n = 8), Ab+ subjects (n = 4), RO T1D subjects (n = 4), relative to Low HLA risk subject group (n = 5), B) fold change of genes uniquely expressed in RO T1D (red bars), Ab+ subjects (dark grey bars) and High HLA risk subjects (light grey bars) relative to Low HLA risk group. C) Genes downregulated in High HLA risk group compared to Low HLA risk group. Cutoff value for presentation of overexpressed genes was >5-fold. * - anti-apoptotic genes.

The analysis of resultant gene expressions between each subject group relative to Low HLA risk (Figure 4A) showed few commonly overexpressed genes across RO T1D, multiple Ab+ and High HLA risk control subjects: Bik, CARD6, caspase2, TNFRSF11B and caspase 14. Tregs from all subject groups overexpressed caspase 2, known as initiator caspase, relative to Low HLA risk. This result is in agreement with our cellular assay showing increased Treg apoptosis in all those subject groups relative to Low HLA risk group suggesting activation of caspase-dependent apoptosis pathway(s). As a highly conserved and tightly controlled process, apoptosis involves activation of many other genes as well. A recent publication indicated the necessity of *BID* expression in the commitment to apoptosis, which is one of pro-apoptotic members of Bcl-2 family [49],. Indeed, compared to Low HLA risk healthy control subjects, both RO T1D and Ab+ subject groups (showing the highest Treg cell apoptosis levels) had increased expression of both *caspase 4* and *BID* genes in Tregs versus Low HLA risk Tregs.

Each of the three subject groups with increased Treg apoptosis also overexpressed a unique set of genes (Figure 4B). Interestingly, some of overexpressed genes among both RO T1D and Ab+ subjects were genes with anti-apoptotic function (RO T1D overexpressed Akt1 (>24-fold), Bag1 (>331-fold) and Mcl-1 (>266-fold) and Ab+ overexpressed BIRC6 (>467-fold), BIRC2 (>14-fold) and Bcl2L1 (>13-fold), suggesting that Tregs are trying to counteract strong pro-apoptotic signal. Additionally, different anti-apoptotic molecules are probably activated at different points in T1D pathogenesis, most likely as a response to different apoptotic stimuli.

*FADD* (adaptor molecule associated with apoptosis) was the only uniquely upregulated gene in High HLA control group when compared to both Low HLA risk and RO T1D (>8-fold and >14-fold, respectively), implying the prevalent Treg apoptosis pathway(s) in this subject group. Interestingly, High HLA risk subject group consists of healthy subjects that have not succumbed T1D despite increased Treg apoptosis. The explanation might lie in apoptosis pathway activated in this subject group. We noticed that many pro-apoptotic genes were overexpressed in both RO T1D and Ab+ subjects (*TNFRSF9*, *TNFRSF10B*, *PYCARD*, *caspases 3*, *7* and *9*, *BIRC8*, *BCLAF1*, *Bak1*, and *APAF1*, Figure 4C). In addition, gene coding *TNFRSF11b* (*OPG*), indirectly involved in apoptosis process, was expressed 34.5-fold more in High HLA risk control compared to Low HLA risk control subjects. This gene acts as decoy receptor for both TRAIL and RANKL [50], and reacts through it to FADD, that was, as mentioned earlier, uniquely overexpressed (>8-fold) in Tregs of High HLA risk subjects, transmitting an inhibitory signal to NFkB and affecting many biological processes. Although OPG was upregulated in RO T1D and Ab+ subjects, lack of FADD overexpression suggests that OPG might in these subject groups react with different ligand potentially increasing Treg apoptosis through different pathways compared to High HLA risk control subjects. Furthermore, it has been shown that inflammatory cytokines elevate OPG expression and release [51]. Intriguingly, OPG is involved in development and function of dendritic cells [52], [53]. All subjects also showed increased expression (52-fold) of *caspase recruitment domain family, member 6 (CARD6)*. This molecule interacts with other family members (like NOD1-CARD4 and Cardiak-Rick or Rip2) suppressing activation of NFkB, but not interfering with their ability to induce caspase1-dependent secretion of interleukin-1b [54]. Deprivation of survival cytokines and the presence of inflammatory cytokines with additional overexpression of Fas/FasL could further increase Treg apoptosis in these groups of subjects as a results of disease progression. Gene expression profile for genes on PCR array done for High HLA risk control group suggests that increased Treg apoptosis is mostly happening via caspase-dependent pathways, more precisely, through involvement of receptors upregulating FADD, but without participation of Fas and FasL, which were downregulated in this subject group when compared to Low HLA control group (<−94-fold and <−17-fold, respectively).

RO T1D Tregs overexpressed pro-apoptotic Bcl2-family genes (>2299-fold Bcl2L10 and >25-fold Bax) Figure 4B), as well as anti-apoptotic genes (>266-fold Mcl1 and >331-fold Bag1) and caspase 14, which might act as an anti-apoptotic molecule due to suppressive nature of its interaction with apoptosis-inducing factor [55]. This suggests desperate attempts Tregs make to recover and counteract already initiated apoptosis process. Uniquely expressed *TRAF2, TRADD and TNFRSF1A* genes in Tregs of RO T1D subjects suggest activation of apoptosis through TNF-family members.

Genes expressed differentially in Ab+ subject group relative to Low HLA risk are presented in Figure 4B. The fact that expression of *caspase 5* was 425-fold higher than in Low HLA risk group suggests that inflammation is substantial at this point of T1D progression. Furthermore, Ab+ subject group overexpresses several pro-apoptotic Bcl2-family members (Bcl2L11 - 17.5-fold, NOD1 – 33.6-fold, Bik – 21.2-fold, Bax – 11.5-fold, Bid – 10.3-fold and Bad – 4-fold) while Bcl2L1 (Bcl2XL, >13-fold), BIRC2 (>14-fold) and BIRC6 (>467-fold) counteracted pro-apoptotic signals. This coordinated overexpression of Bcl2 family members suggests that prevalent apoptotic pathway in Ab+ subjects heavily involve mitochondria. Overexpression of Bcl2L11 (Bim) suggests apoptosis initiation through withdrawal of growth hormones, supporting findings of other studies [36], [56]. Ab+ subjects expressed TNFRSF21/25 (∼15-fold of both), suggesting involvement of TNF pathway to total Treg apoptosis.

Based on gene expression profile of PCR array discussed above supported by *ex vivo* and *in vitro* assays, Tregs of High HLA risk subjects differ from autologous naïve T cells by higher cell turnover completed through a caspase-dependent process that involves molecules transmitting a signal via FADD. On the other hand, analysis of genes expressed in multiple Ab+ subjects suggests that apoptosis is occurring through overexpression of multiple pro-apoptotic members of Bcl2 family, as their dimerization results in changes in mitochondrial membrane potential, release of cytochrome C and imbalance in production of reactive oxygen species (ROS), causing oxidative stress [57], [58]. Oxidative stress has been implicated with both onset and the progression of T1D [59]. Additionally, healthy Ab+ subjects, some of which are already on the path of developing T1D [60], show substantially increased expression of caspase 4 and 5 (>151-fold and >425-fold, respectively), suggestive of an extensive inflammation and overexpression of caspase 2 (>112-fold), whose role was recently expanded to a translational cofactor for DNA damage-induced p21 expression [61].

Overexpression of pro-apoptotic genes from Bcl-2 family was even more pronounced in RO T1D compared to Ab+ subjects (Figure 4A). Literature suggests that production of ROS causes inhibition of complex enzymes of mitochondrial respiration and accumulation of unfolded protein response (UPR) within endoplasmic reticulum (ER) [62], [63]. Increasing body of evidence suggests that cytokines [64], fatty acids [65], [66] and glucose [67], [68] could induce ER and mitochondrial stress, which play an important role in pathogenesis of diabetes at the level of neural, immuno-, and pancreatic beta cells [69], [70]. Mitochondrial and ER stress contribute to increased Treg apoptosis in these groups of subjects. The role of Bcl2 family members (great number of which we have detected) in mitochondrial membrane permeabilization has been proven by their ability to induce cytochrome C release from mitochondria triggering the caspase cascade ending with cell degradation [71]. Additionally, Tregs isolated from RO T1D subjects show increased expression of TRAF2, reported to have an essential role in oxidative stress-induced cell death triggered by ER stress [72], [73]. RO T1D expressed TRAF2 >23-fold and FasL >7-fold compared to healthy Low HLA risk subject group (Figure 4) causing increased transmission of death signal further, most likely via JNK arm activating apoptosis in Treg cells [74].

**Figure 5:**
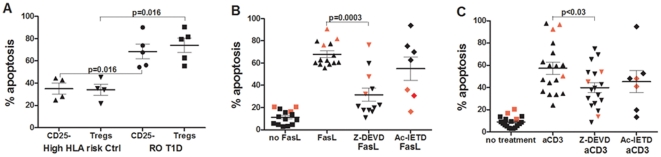
Fas/FasL T-cell apoptosis prevalent in RO T1D, LS T1D and Low HLA risk controls. A) *In vitro* soluble FasL caused significantly lower T cell apoptosis (in both naïve-CD25 and Tregs) in healthy High HLA risk control subjects compared to RO T1D T cells (for both cell subset comparisons Mann-U-Whitney test was p = 0.016), suggesting that Fas/FasL is one of the prevalent apoptosis mechanism in RO T1D subjects, but not in High HLA risk control subjects. B) Treg apoptosis caused by soluble FasL (1/40 dilution – 600 ng/ml) in healthy control, RO T1D and LS T1D subjects was significantly reduced with pretreatment with caspase 3 inhibitor Z-DEVD in majority of subjects. However, analysis of RO T1D subjects alone (red symbols) showed their better responsiveness to pretreatment with Ac-IETD compared to Z-DEVD. C) Activation-induced Treg apoptosis by anti-CD3 was moderately prevented through treatment of both inhibitors, suggesting an involvement of both caspase 3 and 8 in this apoptosis pathway in all subject groups. RO T1D subjects were labeled with red symbols. Mann-U-Whitney test was used for comparisons.

Thus, our results show that in RO T1D and Ab+ subjects in particular, inflammatory processes, mitochondrial and ER stress all converge in caspase - induced apoptosis of Tregs, leading to T1D onset. In Ab+ subjects that have not yet experienced T1D onset, the degree of expression and activation of these genes, which depends on cytokine milieu, will dictate time distance to T1D onset for those subjects, making this period of time particularly important in terms of preventive immunomodulatory treatment. Since our data show that Tregs from both RO T1D and Ab+ subjects die through Fas/FasL pathway, and the opposite was true for High HLA control subjects, we chose this stimulation to validate our microarray and PCR array results. We thus performed *in vitro* cell-based assay where both naïve and Tregs were treated either with soluble FasL (1/40 dilution or 600 ng/ml) for 20 hours (Figure 5A), and proceed further treating Tregs with either soluble FasL treatment or with TCR stimulation, both with and without pretreatment with caspase inhibitors (Figure 5B and C). Knowing that these two apoptosis inductions activate caspase cascade [75], we chose to pretreat cells with caspase 3 (Z-DEVD) and caspase 8 (Ac-IETD) inhibitors. As shown on Figure 5A, manipulated (FasL-treated) Tregs from RO T1D subjects (as well as naïve T cells) were significantly more susceptible to FasL-initiated apoptosis pathways compared to High HLA risk healthy control subjects (Mann-U-Whitney test, p = 0.016, Figure 5A), which was in concordance with our PCR array finding, showing noticeable Fas/FasL downregulation in *ex vivo* Tregs from High HLA risk control subjects (Figure 4A). Studying Tregs isolated from healthy individuals with no consideration of their HLA risk, Fritzsching reported higher sensitivity of Tregs to Fas-mediated apoptosis and lower to that mediated by TCR [76]. In our study, Tregs from healthy Low HLA risk only were responsive to Fas-mediated apoptosis (data not shown), while High HLA risk control subjects showed the opposite apoptotic phenotype, suggesting that HLA might have not yet recognized role in receiving an apoptosis signal. This hypothesis warrents additional studies. In an attempt to abrogate Treg apoptosis in FasL- and the AICD-induced apoptosis pathway, Z-DEVD or Ac-IETD were (or not) used for 30 minutes pre-treatment of Tregs *in vitro* in Low HLA risk controls, RO T1D and Ab+ subject groups (presented together in Figure 5B and C). *In vitro* FasL treatment significantly increased Treg apoptosis through involvement of caspase 3, as apoptosis was significantly lowered by pretreatment with caspase3 inhibitor Z-DEVD in all subjects except in RO T1D subjects (depicted by red symbols throughout Figure 5). Since Treg apoptosis in RO T1D subjects was more efficiently prevented after pretreatment with caspase 8 inhibitor Ac-IETD, quite expectedly showing that the apoptosis pathway in RO T1D subjects was triggered through membrane receptors (Figure 5B). This result clearly suggests more significant involvement of caspase 8 rather than caspase 3 in the FasL-triggered apoptosis pathway in Tregs from RO T1D subjects during honeymoon period. AICD apoptosis activated both caspase 3 and 8 at comparable levels across subject groups.

In conclusion, our data sheds new light on evolving Treg apoptosis pathways and function during T1D development and suggests the importance of HLA in this process. Our results support our hypothesis about the triangular association of HLA risk, Treg survival and Treg function to suggest that different stages in T1D development activate diverse prevalent apoptosis mechanisms, probably as a result of changing surrounding factors impacting further progression towards T1D. This information could be useful when choosing preventive treatment for subjects at different stages of diabetogenesis (either at risk to develop T1D or after T1D onset while in honeymoon phase). Combined with our Treg apoptosis/function methods, apoptosis pathway analysis offers valuable information about potential selection of an immunomodulatory treatment that could prevent further T1D progression in these subject groups. Unquestionably, our results of prevalent Treg apoptotic pathways associated with T1D development warrant further investigation. One type of future study could be a clinical trial employing an agent tailored to block prevalent mechanism(s) in each of the stages of T1D development where our assays would monitor treatment effects in real time.

## Supporting Information

Table S1 * ***DQ2/DQ8*** **heterozygotes are designed as “Very High HLA Risk".**
(DOC)
