# Supplementary material for: Interaction between Treg Apoptosis Pathways, Treg Function and HLA Risk Evolves during Type 1 Diabetes Pathogenesis
Source: PLoS One. 2012 Apr 26;7(4):e36040. doi: 10.1371/journal.pone.0036040 (PMC3338571; doi:10.1371/journal.pone.0036040)
Supplement: Table S1 — * DQ2/DQ8 heterozygotes are designed as “Very High HLA Risk". (DOC) [file pone.0036040.s001.doc]

*Supplementary Table S1: Risk Scheme Details*

|  | | | | | | | |  |
| --- | --- | --- | --- | --- | --- | --- | --- | --- |
| a. Haplotype Risk Assignments | | | b. Genotype Risk Assignments | | | | | c. HLA risk grouping |
|  |  |  | |  |  |  |  |  |
|  | Susceptibilty (S) |  | |  | Genotype |  | Risk Status | HLA risk groups |
|  | 0301-0302 |  | |  | S/S* |  | *Very High* | High HLA risk |
|  | 0501-0201 |  | |  |  |
|  |  |  | |  |  |  |  |
|  | Resistant (R) |  | |  | S/S |  | *High* |
|  | 0102-0602 |  | |  | S/X |  |
|  |  |  | |  |  |  |  |
|  | Weakly Protective (Y) |  | |  | Y/S |  | *Moderate* |
|  | 0102-0603 |  | |  | X/X |  |
|  | 0103-0603 |  | |  |  |  |  |
|  | 0201-0202 |  | |  | Y/Y |  | *Low* | Low HLA risk |
|  | 0501-0301 |  | |  | Y/R |  |
|  | 0201-0303 |  | |  | X/R |  |
|  |  |  | |  | S/R |  |
|  | Neutral (X) |  | |  | R/R |  |
|  | Remaining Haplotypes |  | |  | Y/X |  |
|  |  |  | |  | **DQ2/DQ8 heterozygotes are designated "Very High Risk"* | | |  |
|  |  |  | |  |
